# The Food and Drug Administration and varenicline: should risk communication be improved?

**DOI:** 10.1111/add.13592

**Published:** 2016-10-18

**Authors:** Neil M. Davies, Kyla H. Thomas

**Affiliations:** ^1^ Medical Research Council Integrative Epidemiology Unit at the University of BristolBristolUK; ^2^ School of Social and Community MedicineUniversity of BristolBristolUK; ^3^ School of Social and Community MedicineUniversity of BristolBristolUK

**Keywords:** Adverse drug reactions, evidence based medicine, hierarchy of evidence, nicotine replacement therapy, regulation, varenicline

*Patients and clinicians are likely to interpret the FDA Black Box warning about serious adverse neuropsychiatric adverse events as implying a causal effect of varenicline. This warning does not reflect current scientific evidence accurately and should be revised*.

On 11 May 2006, the US Food and Drug Administration (FDA) approved varenicline (Chantix, Champix) for use in smoking cessation; it was the first oral non‐nicotine treatment licensed by the FDA since bupropion in 1997 1. However, it was not long before concerns were raised about the neuropsychiatric safety of the medicine. Anecdotal reports from popular press and internet sites, in addition to post‐marketing case reports and reports to the FDA's Adverse Event Reporting System (AERS), suggested that some patients prescribed varenicline had experienced suicidal behaviour 2. As a result, in November 2007 the FDA announced that they were conducting two safety reviews of varenicline's associations with suicidality and neuropsychiatric adverse events not related to suicidality 3. Findings from these reviews were published in 2008, with the FDA concluding that ‘[varenicline] may cause worsening of a current psychiatric illness even if it is currently under control and may cause an old psychiatric illness to reoccur’ 4. In July 2009 the FDA went further, mandating that varenicline carry a ‘Black Box warning’ 5. These warnings highlighted ‘the risk of serious neuropsychiatric symptoms in patients using these products’. Symptoms included changes in behaviour, hostility, agitation, depressed mood, suicidal thoughts and behaviour and attempted suicide.

The FDA subsequently commissioned two large observational studies to investigate the potential neuropsychiatric effects of varenicline using observational data from military veterans. Pfizer (the manufacturer of varenicline) was also instructed to undertake a large randomized trial: Evaluating Adverse Events in a Global Smoking Cessation Study (EAGLES) (NCT:01456936). Results from the observational studies were reported in October 2011. In the first, 14 311 patients prescribed varenicline were matched to similar patients prescribed nicotine replacement therapy (NRT) using propensity scores. Patients prescribed varenicline were no more likely than those prescribed NRT to be hospitalized for psychiatric problems [hazard ratio (HR) = 0.76; 95% confidence interval (95% CI) = 0.40–1.46] 3. In the second, 10 814 patients prescribed varenicline were matched to patients prescribed NRT. This study also showed that patients prescribed varenicline were no more likely to be hospitalized for psychiatric problems (HR = 1.14; 95% CI = 0.56–2.34) 6. Observational studies, which can suffer from residual confounding, could not prove that varenicline did not cause neuropsychiatric adverse events. Nevertheless, they provided a more robust form of evidence than the anecdotal and case report evidence used in the original safety review. The FDA ‘determined that the current warnings in the Chantix drug label, based on post marketing surveillance reports, remain appropriate’ and did not remove the Black Box warning 7.

Meanwhile, further evidence was emerging relating to the safety of varenicline. Gunnell and colleagues found no evidence of an increased risk of suicidal behaviour in a cohort of 80 660 patients from the UK's General Practice Research Database 8. In 2013 we updated this analysis to include data from 119 546 patients, using conventional and novel statistical methods to account for residual confounding 9. The findings were the same: there was no evidence that patients prescribed varenicline had an increased risk of fatal or non‐fatal self‐harm. Other observational studies have reported similar findings 10. In 2015 we published a meta‐analysis of results from all available randomized trials, which found that smokers randomized to receive varenicline had similar risks of suicide or attempted suicide to those randomized to receive placebo (OR = 1.67, 95% CI = 0.33–8.57) 11. In March 2015 the FDA issued a further Safety Communication, but did not remove the Black Box warning 12. In 2016 the results of the EAGLES trial was published. This trial randomized 8144 smokers to receive varenicline, transdermal NRT patch, bupropion or placebo, and found that for every 100 smokers allocated to varenicline compared with NRT there were 1.07 (95% CI = –0.08 to 2.21) fewer moderate and severe neuropsychiatric adverse events 13. While it is possible for trials to suffer from bias, this provides the strongest evidence to date that varenicline does not cause neuropsychiatric adverse events 14. Based on the results of this study, the European Medicines Agency (EMA)—Europe's main drug regulator—lifted the warning about possible suicidal risks from varenicline in April 2016 15.

## Black Box warnings, what are they?

Black Box warnings are the strongest warning the FDA issues, and they are ‘designed to call attention to serious or life‐threatening risks’ 3. These warnings must be issued with all prescriptions of the drug in question. The FDA's only escalation after a Black Box warning is to force a drug to be recalled or withdrawn from the market. Black Box warnings indicate either when there is a serious adverse reaction with ‘some basis to believe there is a causal relationship between the drug and the occurrence of the adverse event’ or to ‘highlight important information for prescribers’ 3.

## Correlation does not equal causation

Patients prescribed medications are often at higher risk of adverse events than the general population, even before they start treatment. Therefore, the fact that a medication is associated with adverse events is not necessarily informative for patients. In the case of varenicline, people who smoke are very different from the general population; on average they are poorer, sicker and more likely to have mental health problems 16. Smokers prescribed varenicline recorded in the FDA's AERS were likely to be at increased risk of neuropsychiatric problems before they were prescribed varenicline. The case reports could not take these differences into account in their analysis. Thus, the adverse event reports are likely to be unreliable evidence of the differences in risks of adverse events faced by smokers if they took varenicline or other treatments.

## Should Black Box warnings only be used for causation?

The only information that is useful for patients making treatment decisions is evidence about the causal effects of medications. This should, ideally, be obtained from an adequately powered randomized trial or meta‐analyses of randomized trials. However, there are many instances where randomized trials are not feasible. In the absence of experimental data, regulators should rely upon well‐conducted observational studies. At minimum, these studies should control for a rich set of baseline confounders, use appropriate control groups and avoid or account for as many potential biases as possible 17. Case reports without an adequate control group are unlikely to be sufficient evidence of causation except when the causal effects are extremely large, the adverse event is very rare and the adverse events are not related to the potential confounders 18. However, the incidence of adverse events in treated groups is useful information for prescribers, even if they are not caused by the medication. Should the FDA use Black Box warnings to report information both about the causal effects of medications and patients' baseline risk of adverse events?

## Implications for varenicline

**Figure 1:**
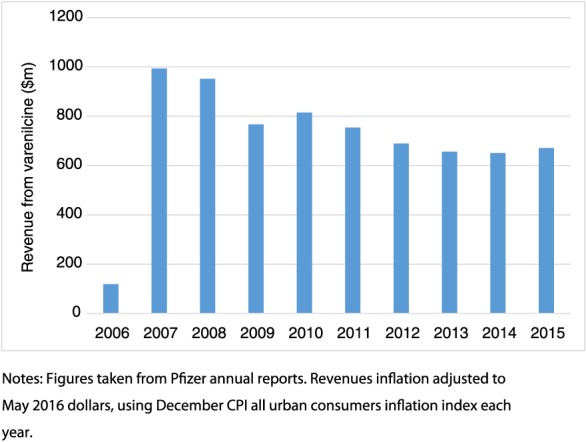
Pfizer revenues from varenicline ($m) by year. [Colour figure can be viewed at wileyonlinelibrary.com]

Effective regulation has to balance all the evidence regarding efficacy and safety, even when evidence is sparse and potentially unreliable. However, false positive signals discovered in case reports can be damaging—the reduced uptake of the measles, mumps and rubella (MMR) vaccine after it was linked (incorrectly) to autism provides a cautionary example in this context. The Black Box warnings issued for varenicline may have confused smokers and physicians about the strength of the scientific evidence about varenicline's neuropsychiatric risks. This is because they may have interpreted the Black Box warning as reporting causal adverse drug reactions. This has serious consequences; fewer people are likely to have been prescribed varenicline as a result of these warnings. Sales of varenicline fell steadily from 2007 to 2014 after adjusting for inflation (Fig. 1). While we cannot be sure how the FDA's warning affected prescribing, the safety concerns may have led to fewer people quitting smoking 18. In addition, Pfizer has been subject to litigation in the United States over potential neuropsychiatric adverse effects of varenicline which they settled out of court at a cost of US$288 million 19. Few may have sympathy for a multi‐billion dollar pharmaceutical company, given the well‐documented misdeeds of big pharma [20]. None the less, Black Box warnings can also impose significant costs on companies developing new treatments, and potentially reduce innovation.

## What should we do now?

From the first warnings about potential neuropsychiatric adverse effects of varenicline in November 2007 to the present, 4.1 million people are likely to have died from smoking‐related disease in the United States alone 16. The EMA has already updated its guidance on varenicline and removed its Black Triangle warnings on varenicline's product labelling. It is time for the FDA to do the same.

## Declaration of interests

None.

Postscript: Since this editorial was first published online, the US Food and Drug Administration has removed the black box warning on varenicline (Chantix). See http://www.medscape.com/viewarticle/873437
